# The effect of structural properties on rheological behaviour of starches in binary dimethyl sulfoxide-water solutions

**DOI:** 10.1371/journal.pone.0171109

**Published:** 2017-02-02

**Authors:** Anna Ptaszek, Paweł Ptaszek, Marek Dziubiński, N. Mirosław Grzesik, Marta Liszka-Skoczylas

**Affiliations:** 1 Agriculture University in Krakow, Faculty of Food Technology, Department of Engineering and Machinery for Food Industry, ul. Balicka 122, Kraków, Poland; 2 Technical University of Lodz, Faculty of Process and Environmental Engineering, Department of Chemical Engineering, ul. Wólczańska 175, Łódź, Poland; 3 Institute of Chemical Engineering, Polish Academy of Science, ul. Bałtycka 5, Gliwice, Poland; North China Electric Power University, CHINA

## Abstract

This research study analysed the rheological properties of potato amylose and potato amylopectin in binary solutions of the following water and dimethyl sulfoxide concentrations: 90% DMSO (1), 80% DMSO (2) and 50% DMSO (3), with preparation methodology involving the dissolution at the temperature of 98°C. The studies of dynamic light scattering on the biopolymer coils and the determination of main relaxation times of the solutions were carried out. For the amylose solutions, the fast relaxation phenomena are predominant. The results of the quality tests of the hysteresis loop showed, that the amylose solutions in the solvents (1) and (2) are rheologically stable and shear-thickened. The amylose solutions in solvents (3) reveal oscillatory alterations of viscosity in the time. Amylopectin solutions are characterized by 80% share of slow relaxation phenomena, very low diffusion coefficients and hydrodynamic radii in the range of 2000 nm. The amylopectin solutions are rheologically unstable.

## Introduction

The structure-forming properties of starch are a result of its composition, and the interactions between its polysaccharide chains and the solvent’s molecules. There are only few known systems in which starch can dissolve [1], i.e., in water, dimethyl sulfoxide (DMSO) and N,N-dimethyloacetamide supplemented with LiCl. Dissolution of starch requires a total destruction of the starch granule, which is composed of layers of crystalline structure, as well as amorphous layers. As a result, a solution containing both linear and branched chains is obtained. The chains’ conformation, as well as their behaviour, are distinct and depend on the type of the solvent. Amylose (AM), which in aqueous solutions adopts a helix conformation, undergoes a process of gelation immediately after it is released from a granule; the gelation process begins upon crossing the c* concentration (overlap concentration) [2, 3]. Amylopectin (AP) chains adopt a coil conformation in water; the coils form a network due to the entanglement of the side branches. The values of average radii of gyration and weight average molecular mass obtained in the experiments with static light scattering (SLS) [4] and with the help of chromatography studies, are comparable [5] and indicate, that amylopectin assumes a spherical or globular conformation in DMSO, whereas amylose adopts a conformation of an elastic chain.

A mixture of DMSO and water can be used as a special solvent. The aqueous solutions of DMSO are still explored due to their unique properties. These solutions exhibit a strongly non-ideal behaviour, due to interactions between molecules. The methyl groups of DMSO may induce cooperative ordering in the system by hydrophobic hydration effects. The oxygen atom of a DMSO molecule can interact with water through H-bonding [6]. Roy and co-workers [6] observed the continuum percolation transition in aqueous DMSO solutions with the percolation threshold of 12–15% wt. of DMSO. As a consequence of the specific nature of DMSO-water interactions, certain anomalous physical properties are observed for linear hydrocarbon chains, [7] as well as some proteins such as lysozyme [8, 9], in binary solvents at low DMSO concentrations.

In the case of starch, a number of studies have been carried out over the years to understand the conformational properties of linear amylose [10– 14] and branched amylopectin [14–17], in water and DMSO mixtures. These studies focused, for the most part, on the conformational transformations of AM or AP in a binary solvent at high DMSO concentrations (90% v/v). Han & Lim [14] studied the influence of the conditions of dissolution in 90% DMSO on the molecular parameters of starch with varying amylose content. The authors determined, that the temperature and time of the dissolution have substantial influence on the weight average molecular mass, radius of gyration and the dispersion of the polysaccharide chains in the solution. With the increasing addition of water, the interactions between amylose and DMSO were reduced, leading to the conformational transition of AM from tight helical via loose helical to disordered coil [11]. This led to a decrease of the value of radius of gyration (R_g_) [10], the changes to AM’s weight average molecular masses [12] and reduction of the intrinsic viscosity values [11, 12]. Moreover, at the concentration of 65% DMSO, a formation of DMSO·2H_2_O hydrate and the solvation of amylose were observed [11]. Lee, You, Kweon, Chung, & Lim [17] studied the solubility of waxy maize starch in binary solvents (DMSO/H_2_O: 500/500, 700/300 and 900/100) and determined, that the AP’s solubility is limited by the presence of water in the solvent. An increasing water content not only limits the AP’s solubility, but also causes an aggregation of its chains in the solution. These observations were confirmed by the results presented in the study by Vasconcelos et al. [13]. In the case of starch solutions in binary solvents, the phenomenon of coil overlap occurs, observed previously for water solutions of amylopectin [2, 3].The test results indicate the presence of quite strong polymer-polymer type interactions. In the case of potato starch dissolved in a (DMSO/H_2_O 800/200 v/v) solution, the overlap concentration equals c* = 0.015 g·mL^-1^, and intrinsic viscosity equals 0.5 dL·g^-1^ [13].

A separate group of studies concerned the rheological properties of starch solutions in binary solvents, and discussed the properties of rheologically unstable solutions of cornstarch in 90% DMSO [18, 19]. Additionally, several studies on viscoelastic properties of solutions of maize amylopectin in 90%DMSOwere carried out [20– 22]. The authors determined that the properties of solutions of branched amylopectin depend on its concentration; moreover, they are solutions of a non-Newtonian nature. The solutions of amylose in 90% DMSO studied in the research by Kapoor & Bhattacharya [21, 22] behaved like Newtonian liquids.

The results of tests on structural properties of biopolymers in binary solvents, and rheological properties of their solutions in 90% DMSO, presented in the research literature, point to a relation between the conformation of polysaccharides, adopted in the solutions, and the behaviour of those solutions during shear flow.

The above considerations indicate, that the body of knowledge concerning rheological behaviours of starch and its components (amylose and amylopectin) in binary water/DMSO solutions is rather fragmented and certain phenomena are not yet fully described. There is a lack of a clear description of thixotropic behaviours, as well as behaviours occurring during long lasting shearing. Due to a very complex mechanism of water/DMSO/starch interactions, shear-induced conformational changes can occur in such systems. This in turn can lead to the loss of stability of flow, manifested by a physicochemical catastrophe of a static or dynamic kind, and the occurrence of non-fading viscosity oscillations in time. Moreover, there is a lack of equations describing the dependencies of viscosity and shear rate in the subject literature, resulting from the fact that these solutions do not comply with simple correlations of the Ostwald-de Waele equation, and require the use of complex models and special methods of parameter estimation. There is also a notable absence of cross-referencing of the results of DLS studies in conjunction with molecular structure of the discussed solutions, with the results obtained from rheological studies.

The aim of this study was therefore the analysis of nonlinear rheological properties of the binary solutions (water/DMSO) of potato amylose and amylopectin. Particular emphasis was placed on the interpretation of the thixotropic behaviours and phenomena taking place during long-term shearing. The obtained results were compared with the data from research literature, as well as with data obtained from the studies on dynamic laser light scattering in the analysed solutions.

## Materials and methods

The research materials comprised solutions of starch (1% and 5% w/w) in a binary solvent, formed of water and DMSO at various ratios [10]. Pure potato amylose (Sigma, Germany) and pure potato amylopectin (Eliante Avebe, the Netherlands) were used in this research.

### Gel chromatography GPC

Approximately 20 mg of the analyzed sample was weighed with accuracy of 0.1 mg and placed in a tube; next, 2 mL of 0.1 mol·L^-1^ NaOH was added and the content was mixed until the compounds became diluted. A drop of phenolphthalein was added as a marker and subsequently neutralized with 2 mL of 0.1 mol·L^-1^ HCl. Next, the sample was filtrated and injected into a system of chromatographic columns. The chromatographic analysis of the starch samples was carried out using gel chromatography (GPC).

The system consisted of two columns connected in a series (Ultrahydrogel 500, Ultrahydrogel 2000—Waters). A solution of 0.1 mol·L^-1^ NaNO_3_ and 0.02% NaN_3_ was applied as an eluent. The flow rate was set to 0.6 mL·min^-1^ and 100 μL of the sample volume was injected. Calibration was performed using the pullulan (Shodex) standards.

The following values were obtained for amylose: weighted molecular mass M_w_ = 35 kg·mol^-1^, number molecular mass M_n_ = 23 kg·mol^-1^, polydispersity 1.6, and for amylopectin respectively: M_w_ = 35 710 kg·mol^-1^, M_n_ = 18 650 kg·mol^-1^, polydispersity 2.

### Preparation of solutions of starch in mixtures of H_2_O and DMSO

As described in the reference literature, the ratios between water and DMSO (v/v) were maintained as follows (1) 100/900, (2) 200/800, (3) 500/500 and (4) 700/300. The influence of the sample preparation procedure on the properties of the sample was studied. Consequently, a series of experiments were conducted; samples were prepared based on the following three different protocols:

Method I: starch was diluted in binary solvents at 23°C.

Method II: starch was diluted in binary solvents at temperatures varying from 98°C.

Method III: starch was subjected to pasting in a fixed amount of water at 98°C; next, DMSO was added until the concentration of the mixture reached the level of the concentration of the binary solvent; the solution was mixed at the same temperature.

The solutions were mixed at the appropriate temperatures for 24 hours [14, 23].

### Rheological measurements

Rheological measurements were made using an RS 6000 rheometer (ThermoFischer, Karlsruhe, Germany). A sensor system of a cone-plate type was used, with the parameters set as follows: d_in_ = 35 mm at the angle of 2°. In the first phase of the experiment the setting of the sensor was optimized, which eliminated its effect of inertia when carrying out the viscosity measurements proper. Next, the rate, with which the sensor was introduced to the sample, was adjusted in order to obtain repeatability of the results. The study was undertaken at 23°C. The temperature was controlled by the ultra thermostat, with the accuracy of 0.10 K.

### Flow curves

Prior to the 10-minute measurement program, the sample was subjected to relaxation (under the sensor) for 10 minutes. In the first phase of the measurements, the $\dot{\gamma }$ shear rate was increased to 1000 s^-1^ (the “up” curve) within 5 minutes. Once the desired $\dot{\gamma }$ value was reached, the measurements of the apparent viscosity were made, under the conditions of decreasing shear rate (to zero) within the same period of time (the “down” curve). The measurements were repeated three times. The study was carried out at 23°C.

### Equilibrium measurements

This type of experiment comprised the measurements of changes of the apparent viscosities, within 5 minutes at a fixed shear rate. This measurement procedure was chosen due to complex rheological behaviour of the polysaccharides’ solutions [24, 25]. It allows to recognize for the detection of the typical thixotropic behaviour in a studied sample in a wide range of time-dependent rheological properties [26–28]. The following measurement conditions were applied: 12 selected shear rates: $\dot{\gamma }$: 0.5 s^-1^, 0.7 s^-1^, 1 s^-1^, 5 s^-1^, 10 s^-1^, 20 s^-1^, 50 s^-1^, 70 s^-1^, 100 s^-1^, 200 s^-1^, 500 s^-1^ and 1000 s^-1.^ The measurements were carried out at 23°C.

### Intrinsic viscosity

The starch solutions were subjected to specific viscosity measurements, using a system comprised of Ubbelhode’s viscometer (SI Analytics, Germany) with capillary constant (K) equal to 0.01 mm^2^·s^-2^, electric clock ViscoClock, (SI Analytics, Germany) and a water bath (CT52, SI Analytics, Germany). The measurements were made at the temperature of 23°C. As a first step, viscous properties of the starting solutions of H_2_O and DMSO were determined. Next, the AP solutions in binary solvents were analyzed. The viscosity η of the solutions was calculated using the capillary constant (K). The method of analysis was based on determining the intrinsic viscosity [η] and Huggins constant K_H_ from the equation [29]:

$$\eta_{red}=\frac{\eta_{sp}}{c}=\left[\eta \right]+K_{H}\cdot {\left[\eta \right]}^{2}\cdot c$$ (1)

where $\eta_{sp}=\frac{\eta }{\eta_{sol}}-1$, η_sol_ is the viscosity of the binary solvent and c is given in g·mL^-1^. All calculations were carried out using the nonlinear minimization algorithm of Marquardt-Levenberg.

### Dynamic light scattering measurements

Dynamic light scattering measurements were carried out at 23°C on a Brookhaven DLS/SLS BI-160 goniometer with digital autocorrelator BI-9000AT (Brookhaven, New York, USA). A solid-state laser (JDSU, CDPS532M-050) with output power 50 mW at λ = 532 nm was used as a light source. The studies on the solutions’ properties were performed at a scattering angle θ of 90°. The time–average intensity correlation functions g^2^(τ)-1 were obtained with an acquisition time of 300 s for each run, with the help of the Brookhaven Instruments Dynamic Light Scattering Software. The mathematical analysis was based on the fitting of Kohlrausch-Williams-Watts stretched exponential function [30] to the correlation functions g^2^(τ)-1. The values of relaxation times of the fast τ_f_ and slow τ_s_ components as well as fractional contribution of the fast process (A) and the exponent of the stretched exponential (β) were estimated with the help of Marquardt-Levenberg minimization method. Next the values of hydrodynamic radius R_h_ and diffusion coefficient D_f_ for fast modes were calculated.

### Modelling and estimation of the rheological equation parameters

The equation of state may be applied in the description of the rheological properties of starch pastes in a form, which corresponds to the shear-thinned systems [31]:

$$\eta_{app}\left(\dot{\gamma }\right)=\tau_{0}\cdot {\dot{\gamma }}^{-1}+\sum_{p=1}^{\infty }\eta_{p}\cdot e^{-t_{p}\cdot \dot{\gamma }},$$ (2a)

or to the shear-thickened systems [32]:

$$\eta_{app}\left(\dot{\gamma }\right)=\tau_{0}\cdot {\dot{\gamma }}^{-1}+\sum_{p=1}^{\infty }\eta_{p}\cdot \left(1-e^{-t_{p}\cdot \dot{\gamma }}\right).$$ (2b)

The abbreviations used in the equation are as follows: η_app_ means the apparent viscosity (Pa·s), τ_o_ stands for the yield stress (Pa) and $\dot{\gamma }$ represents shear rate (s^-1^). The symbols: t_p_ and η_p_, p = 1,2,… are the parameters of the equation of state and they can be interpreted as the corresponding time constant(s) and its intensity (Pa·s), respectively [32].

An estimation of parameters of only one addend in the sum (Eq 2), i.e. the estimation of the t_1_ and η_1_, facilitates the determination of the so-called most probable characteristic time (t_1_). However, a comprehensive description of the system is not possible in this case.

As the number of estimated parameters increases, this problem becomes computationally more complex (ill-posed), which in turn makes the application of the least squares method impossible. The regularization method [33] was applied to estimate the parameters in the equation of state.

The intensity values η_p_ are dependent on the values of apparent viscosity. The higher the apparent viscosities, the higher the values of this parameter become. In order to compare the results for the systems of diverse concentrations and of diverse viscosity resulting from this, a normalization of the intensity value η_p_ against the maximum value η_max_ in the given distribution η_p_/η_max_ was carried out. As a result of the implementation of this procedure, the intensities of all characteristic times are located within the range from 0 to 1 and the shape of the distribution and the location of the maxima on the graph are maintained.

For comparison, the parameters of the most common power-law equation (Herschel-Bulkley model) were estimated. The following objective function:

$$\chi_{M-L}^{2}=\sum_{j=1}^{N}{\left(\eta_{j}^{\delta }-{\hat{\eta }}_{j}^{}\right)}^{2}\to \underset{\tau_{o},k,n\geq 0}{\min}$$ (3)

where: ${\hat{\eta }}_{j}^{}=\eta \left({\dot{\gamma }}_{j}\right)=\tau_{0}\cdot {\dot{\gamma }}_{j}^{-1}+k\cdot {\dot{\gamma }}_{j}^{n-1}$ was minimised using the Marquardt-Levenberg method. The value of dissipated energy ΔE was calculated numerically with the help of the trapezoidal method.

## Results

The dissolution of potato amylopectin, or amylose, in the mixture of DMSO and H_2_O in the ambient temperature turned out to be ineffective (Method I). The values of the second osmotic virial coefficient, determined for the solutions of the analyzed starches in pure DMSO and in water at 30°C are low [34]; this explains difficulties in dissolution of starch in lower temperature conditions. Only when the temperature increased to 100°C, the solutions became clear (Method II).

**Fig 1:**
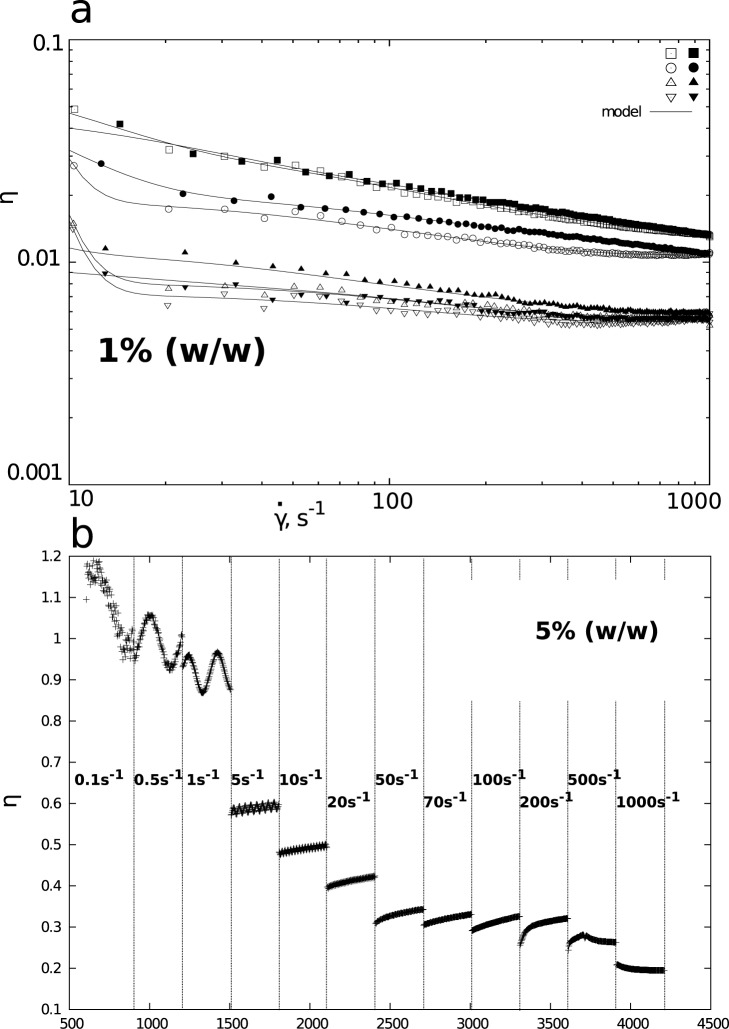
Apparent viscosity dependence on a) shear rate for 1% (w/w) amylopectin solutions in binary solvents (open symbols represent “up” flow curve, filled “down” flow curve) and b) time of shearing at selected shear rates for 5% (w/w) amylopectin solution in solvent (3). Results shown for binary solvents H_2_O/DMSO: (1) 100/900, (3) 500/500 and (4) 700/300.

Certain interesting properties have been observed for the amylopectin (AP) solutions (Fig 1). The AP solution in the solvent (1), i.e. in a mixture of H_2_O/DMSO (in the ratio of 100/900; symbol II (1)), showed the highest apparent viscosity. The solution undergoes shear thinning within the full range of shear rate (Fig 1A). Moreover, the phenomenon of hysteresis of the “up” and “down” curves disappears with the increasing shear rate.

The AP solution, prepared in a mixture of water and DMSO in the proportion of 500/500 (symbol II (3)), was characterized by lower apparent viscosity, while simultaneously retaining its shear thinning properties. However, in this case a clear hysteresis loop may be observed. The “down” curve runs above the “up” curve; this fact points to the accumulation of mechanical energy in the solution. The hysteresis of the flow curves is visible in two other solutions (Table 1). The first solution (symbol II (4)) was obtained by dissolving AP in a binary solvent (99 g of a solvent, 98°C), whereas the second one (symbol III, (4)) was obtained by pasting 66.87 g of AP in 67 mL of water at 98°C; next, 31.57 g (28.7mL) of DMSO was added and stirred under the same temperature conditions. In both cases the phenomenon of hysteresis is observed (Table 1).

**Table 1:** The values of the parameters of the model (2a) for 1% AP solutions and of the model (2b) for 1% AM solutions (sample volume 0.4∙10^−6^ m^3^).

Starch		Up					down					ΔE,J·10^6^
		Herschel-Bulkley		time constants (Eq 2)			Herschel-Bulkley		time constants (Eq 2)			
		K	n	t_1_, s	t_2_, s	t_3_, s	k	n	t_1_, s	t_2_, s	t_3_, s	
AM	II(1)	1.93·10^−5^	1.08	0.1	5·10^−4^	-	1.93·10^−5^	1.08	0.1	5·10^−4^	-	-
	II(2)	2.07·10^−5^	1.10	10	10^−3^	-	2.07·10^−5^	1.10	10	10^−3^	-	-
	II(3)	1.05·10^−5^	1.25	10^−5^	-	-	1.05·10^−5^	1.25	10^−5^	-	-	-
AP	II(1)	0.066	0.77	0.1	0.02	0.005	0.065	0.77	0.05	0.005	5·10^−4^	-2.68
	II(3)	0.031	0.84	0.7	-	-	0.046	0.79	0.7	-	-	-2.78
	II(4)	0.013	0.87	0.7	-	-	0.034	0.71	0.5	0.02	0.002	-1.40
	III(4)	0.011	0.87	0.7	-	-	0.011	0.89	0.1	0.007	-	-1.19

The second graph (Fig 1B) illustrates the results of the equilibration experiment carried out for the 5% (w/w) AP solution. Within the range of lower shear rates (up to 10 s^-1^), oscillatory changes of viscosity in time can be observed; the amplitude of the changes decreases as the shear rate grows. The value of the amplitude (viscosity oscillations) drops as shear rate grows; this is due to the fact that the system receives increasing amounts of energy, which causes the newly obtained structure to become loose (viscosity decreases). Hence, the accumulation of energy in the structure occurs promptly and the system achieves boundary values (the maximum) within a very short time; consequently, the system also rapidly dissipates energy. The emerging structures, corresponding to individual shear rates, exhibit a weaker nature (the value of viscosity decreases). However, the change of viscosity in time is increasing, i.e. the system tends towards a stationary state and creates a structure that strongly resists the flow (it undergoes shear-thickening). This phenomenon is observed up to the value of $\dot{\gamma }=500s^{-1}$, which is accompanied by changes in the system’s properties and the decrease in viscosity. This implies a disintegration of the structure or its reorientation against the direction of the flow.

Molecularly, a solution is an environment where new structures are both formed and degraded; however, their description requires a combining of methods, e.g. optical methods with simultaneous rheological measurements.

**Fig 2:**
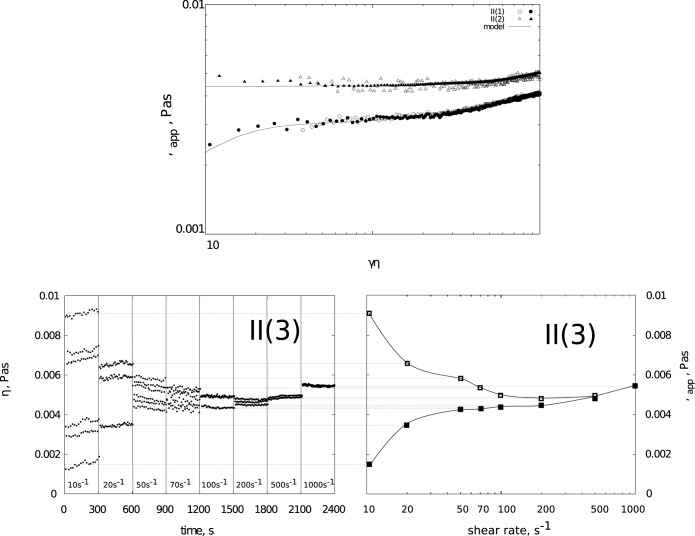
a) Apparent viscosity dependence on shear rate for 1% (w/w) amylose solutions in binary solvents (open symbols represent “up” flow curve, filled “down” flow curve). Results shown for solutions in binary solvents H_2_O/DMSO: (1) 100/900 and (2) 200/800, b)Amplitudes of viscosity in time series as a function of shear rates (left) and possible courses of flow curves (Figenbaum-like diagram, right). Results shown for 1% (w/w) amylose solution in solvent (3) H_2_O/DMSO: 500/500.

Fig 2A shows the flow curves for the AM solutions prepared according to the method II (1) and II (2). The systems do not exhibit a visible hysteresis (Table 1) and their rheological behaviour is less complex. Moreover, the values of the apparent viscosity are more than two orders of magnitude lower in comparison to amylopectin. Both systems show the phenomenon of shear thickening within the range of higher shear rates.

The solution of AM in the 500/500 H_2_O/DMSO solvent (II (3)) exhibits completely different behaviour. Fig 2B presents time series, which illustrate the changes of viscosity in time, in a function of incrementally-increasing shear rate of the AM solution prepared under the conditions II (3). Within the range of lower shear rates, the viscosity fluctuates in an oscillatory manner; the changes, however, are very complex. The graph illustrates solely the extreme values of viscosity. In the case of shear rates of the order of 10 s^-1^, the course of the η(t) is characterized by such complex oscillatory behaviour, that six extreme values of viscosity are observed.

The rise in shear rate causes the rheological behaviour of the solution to undergo certain changes. The alteration in extreme values ($\dot{\gamma }$ = 20 s^-1^ and $\dot{\gamma }$ = 50 s^-1^) is observed; however, for $\dot{\gamma }$ = 70 s^-1^ the changes are likely to occur in a chaotic manner. Further increase in shear rate is accompanied by a visible reduction of the oscillatory amplitude. Moreover, a noticeable rise of viscosity in the function of shear rate can be observed. The presented graph may be regarded as a Feigenbaum-like diagram, which uses shear rate as a bifurcation parameter [35]. As a result of the aforementioned changes, the analyzed solution II (3) can be illustrated by different courses of the flow curves. The internal graph depicts two variants of the alterations of apparent viscosity in the function of shear rate (classical flow curve). The curve spotted with black squares represents the shear-thickening phenomenon. The measuring points, which create the curve, mirror the values of the amplitude of viscosity ranging from 0.001 to 0.005 Pas; i.e. the values are minimal.

The course of the second, alternative flow curve presented in Fig 2B, within the range of lower shear rates, corresponds to shear-thinning. The points, which are marked with grey squares, result from amplitudes ranging from 0.006 to 0.01 Pas, visible on the graph.

## Discussion

The rheological properties of AP and AM solutions in binary solvents are quite different from each other. In the case of AP solutions, certain characteristics—defined as time-dependent—can be observed. The AM solutions behave as shear-thickened, while AP solutions produce shear-thinned systems. Moreover, amylose solutions are characterised by decidedly lower apparent viscosity, compared to the corresponding amylopectin solutions.

**Fig 3:**
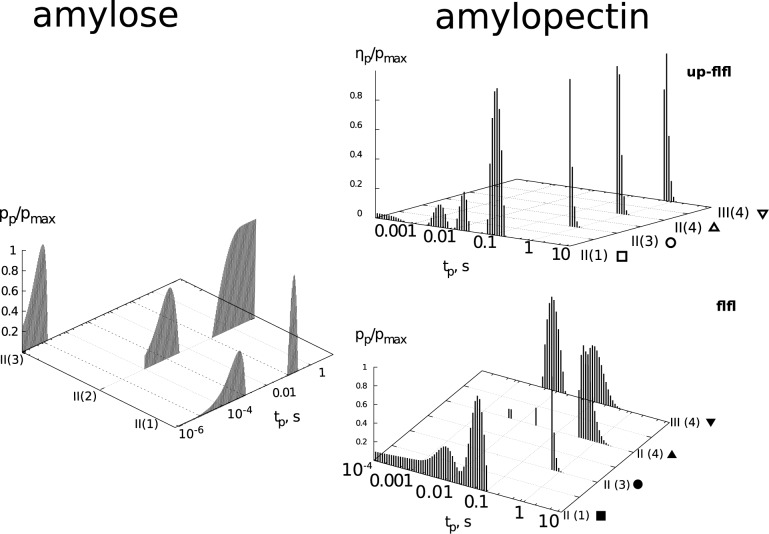
Characteristic times distributions for shear-thickened solutions of amylose and rheounstable shear-thinned amylopectin solutions (distributions for up- and down-flow curve).

Among the group of amylose solutions, the lowest apparent viscosity is exhibited by AM solutions in solvent (1). This phenomenon might be attributed to the molecular characteristics of AM in binary solvents (Table 2). The study literature indicates the radius of gyration (R_g_) of amylose, dissolved in binary solvents with 10% water content, to be in the 37.5 nm range, reaching the maximum value of 38.8 nm for solutions with 20% water content, and subsequently decreasing to the level of 26.3 nm for solvents with 70% water content (Table 2). The studies of aqueous solutions of AM, in turn, demonstrated the radius of gyration for this polysaccharide from cereal grain to be 25 nm [2, 3] and for potato amylose—62.5 nm [36]. By comparing these datasets, it could be concluded that in solutions of up to 40% H_2_O content, amylose adopts a helix conformation [37]. For the discussed solution (1), the average R_h_ value determined in the DLS tests equals 780 nm (Table 3), and indicates that the diffusion of AM chains in the solution is strongly limited by the biopolymer-solvent interactions. The comparison of the properties described by the R_g_ value from (Table 2) with the results of DLS measurements suggests (S1 Fig), that the conformation adopted by AM in solution (1) prevents it to diffuse freely. The participation share of the fast and slow relaxation phenomena, determined based on the Kohlrausch-Williams-Watts model indicate, that relaxation is in 60% driven by the fast processes (Table 3). This might signify, that despite large sizes and low translational diffusion coefficient, in the range of lower shear rates, the AM chains are capable of directional change along the stream lines, caused by laminar flow. This hypothesis would explain the low apparent viscosity of the solution in the low shear rate ranges (< 100 s ^- 1^). Further increase in shear rate results in an increase of apparent viscosity (Fig 2A), most probably as a result of high resistance the chains exhibit during the flow. In these flow conditions, the limited diffusion properties become evident. It is also possible, that the chains undergo deformation during shear flow, due to high hydrodynamic radius (R_h_) values and the helix conformation assumed in this solution. The values of time constants (Eq 2b, Fig 3, Table 1), determined for the discussed flow curve (Fig 2A), delineate a distribution with two peaks. The maximum of the first peak corresponds to the characteristic time of 0.1 s, while the maximum of the second peak is 5·10^−4^ s. The presence of two groups of time constants could be related to the presence of two types of phenomena, registered with the use of DLS technique: fast and slow, represented by two relaxation times t = 0.2 μs and 3600 μs (Table 3).

**Table 2:** Molecular parameters of starches in binary solutions H_2_O/DMSO at 25°C.

starch		Solution	M_w_,	A_2_,	R_g_,	R_h_,	[η],	α^2^	ρ	
		H_2_O/DMSO	kg·mol^-1^	mol∙mL∙g^-2^	nm	nm	mL·g^-1^	-	-	
AM	potato	100/900 → (1)	765±224	(272±77)×10^−6^	37.5±2.0	-	157±0.8	2.192	-	[10]
		200/800 → (2)	660±67	(276±25)×10^−6^	38.8±2.0	-	143±0.7	1.952	-	
		500/500 → (3)	555±44	(123±9)×10^−6^	34.0±1.8	-	83.4±0.4	1.304	-	
		700/300 → (4)	506±38	(55.6±36)×10^−6^	26.3±1.4	-	65.7±0.3	1.189	-	
	maize	0/1000	-	-	-	-	120	-	-	[11]
		340/660	-	-	-	-	60	-	-	
		500/500	-	-	-	-	40	-	-	
		670/330	-	-	-	-	25	-	-	
		100/900 → (1)	151±1.5	-	84±0.3	-	-	-	-	[14]
AP	maize	100/900 → (1)	(171±1.4)×10^3^	-	238±0.3	-	-	-	-	[14]
		100/900 → (1)	150×10^3^	5.5·10^−8^	238	190	-	-	-	[16]
		100/900 → (1)	(15.3±1.4)×10^3^	-	99.8±5.5	107.0±5.9		-	0.91±0.11	[17]
		300/700	(57.5±10.3)×10^3^	-	182.3±25.8	173.5±8.6		-	1.06±0.19	
		500/500 → (3)	(192.7±14.1) ×10^3^	-	182.8±10.9	201.2±19.0		-	0.93±0.06	
		100/900 → (1)	-	-	-	-	80–220	-	-	[37]
	potato	0/1000	-	-	-	-	178±1	-	-	[38]
		100/900 → (1)					38±2			this work
		200/800 → (2)					92±2			
		500/500 → (3)					117±5			
		700/300→ (4)					102±9			

**Table 3:** Parameters of Kohlrausch-Williams-Watts equation estimated for 1% solutions of amylose and amylopectin in binary solvents.

		τ_f_, μs	τ_s_, μs	A	D_f_, cm^2^·s^-1^	R_h_, nm
AM	II (1)	0.2	3600	0.6	8.8·10^−11^	780
	II (2)	0.1	3700	0.6	2.1·10^−10^	290
	II (3)	0.2	3700	0.5	7.3·10^−11^	760
AP	II (1)	500	3300	0.2	3.4·10^−14^	2050
	II (3)	200	2400	0.2	8.1·10^−14^	1750
	II (4)	700	2700	0.2	2.5·10^−14^	2240

The next analysed amylose solution II(2) is characterised by slightly higher apparent viscosity (Fig 2A); however in this case the strong dependency of apparent viscosity on the shear rate is not observed. Similar behaviour has been noted for high amylose cornstarch solutions (70% of amylose) and described in the study [21]. The authors have determined, that 2% (w/v) solutions of this starch in 90% DMSO behave as Newtonian liquids of 18 mPas viscosity. As research studies indicate [11], in solution (2) the amylose chains assume a loose helix conformation, and are characterised by the highest radius of gyration (Table 2). The dynamic light scattering studies have indicated, in turn, that the AM chains in solution (2) are characterised by the highest value of the diffusion coefficient D_f_ (Table 3) and the smallest hydrodynamic radius. In the case of AM solution II (2) the value of R_h_ is at the 290 nm level (the lowest in Table 3), while, for the same solution, the amylose chain is described by a high value of R_g_ (Table 2). In all probability, this is the reason the solution’s rheological behaviour does not visibly change along with the shear rate. The time constants (Eq 2b, Fig 3) delineate a distribution graph with two separate peaks (t_1_ = 10 s and t_2_ = 10^−3^ s), wherein the peak with a maximum of 10 s does not disappear. The most interesting rheological behaviour was observed in the case of solution II (3) (Fig 2B). As previous research indicates, the AM chains in solution (3) can assume a conformation intermediate between a loose helix and a coil [10, 11]. The value of the diffusion coefficient, determined in the course of DLS tests, is the lowest (7.3·10^−11^ cm^2^·s^-1^) and the hydrodynamic radius is in the 760 nm range (Table 3). These parameters are similar to those obtained for the solution II (1). The increase of the apparent viscosity (Feigenbaum-like diagram, Fig 2B right, the flow curve corresponding to shear thickening) is equivalent to the II (1) solution. However, the alternative course of a part of the flow curve (Fig 2, grey points), resulting from oscillatory changes in viscosity (Fig 2B left), indicates that the chains are capable of forming such structure in the solution, that exhibits high resistance during flow. This is possible due to a loose structure of the AM chain in a solution, in which water constitutes 50%. The discussed phenomenon is visible only in the range of low shear rates and disappears with the increase of $\dot{\gamma }$. Similar irregular oscillating behaviour was observed in the case of carrageenans water solutions [39], as well as during the shearing of amylopectin pastes [34].

The solutions of AP constitute shear-thinned systems. Similar behaviour is exhibited by solutions of corn amylopectin dissolved in 90% DMSO [21]. In the case of the 1% potato amylopectin solutions (Fig 1A), the flow curves hysteresis can be observed. This phenomenon is a result of the solutions’ capability to accumulate mechanical energy (Table 1). The shear-thinning phenomenon is possibly linked to the structure of AP in the solutions (Table 2, ρ values). As previous research indicates [17], amylopectin in binary solutions assumes a regular star structure. Its average hydrodynamic radius decreases with the increase of DMSO content in the aqueous solvent [17]. With the increase of water content in the binary solvent, the share of AP chains with hydrodynamic radius over 1000 nm increases in particle size distribution [17]. This phenomenon can be explained by amylopectin molecules being less dispersed, existing as larger particles, which would suggest the possibility of aggregation [17]. This effect is illustrated by the values of intrinsic viscosity determined in this study. In the case of solution AP II(1), the [η] = 38 mL·g^-1^, while for solution II(3) it is [η] = 117 mL·g^-1^. The results of DLS tests (Table 3) indicate a small share of fast components (20%) in the shaping of relaxation phenomena. Slow relaxation times are in the 3000 μs range, while fast relaxation times are significantly higher, than in the case of amylose solutions, and range from 200 to 700 μs. The values of R_h_ are in the range of 2000 nm, with the highest value of 2240 nm corresponding to solution II(4). In the case of all studied AP solutions, the values of diffusion coefficient are very low (Table 3). Therefore, the chains present in 1% solutions form a stable structure [17], in which the chains of branched macromolecules of regular star structure rub against each other during the flow, which is imposed by the external mechanical forces field. An increase of AP’s concentration in solution II(3) to 5% (Fig 1B) causes not only an increase in viscosity, but also a formation of oscillatory phenomena (Fig 1B). They might be related to amylopectin’s tendency to aggregate in solutions with high water content [17]. The distributions of time constants (Table 1, Fig 3) indicate, that the solutions’ rheological behaviour, in conditions of shear rate reduction (curve “down”), is more complex than the properties described by the curve “up”. In the case of 1% solution II(3), a noticeably higher apparent viscosity is visible on the “down” curve (Fig 1A). This phenomenon, together with the time-dependent behaviour for the 5% solution II(3) (Fig 1B) might suggest, that AP chains of very large R_h_ and loose structure [13] are capable of accumulating mechanical energy. This might be related to slow chain relaxation (Table 3, S2 Fig)), which prevents the release of mechanical energy in a short time span. Because of that, the “down” flow curve also runs above the “up” curve. An increase of the AP in the solution results in certain rheological phenomena being revealed [21, 22]. For the solution of 5% concentration (Fig 1B), oscillatory changes of viscosity in the lower shear rate range were observed, together with shear thickening. Similar behaviours have been observed for corn amylopectin dissolved in 90% DMSO [18, 19]. The authors of the quoted study linked these behaviours to the formation of a structure by the AP chains, during shear flow. In the course of the “up” flow curve, a formation of a structure in the liquid, or chain orientation, takes place. During the second “down” cycle, the solution behaves as a liquid with an already-formed structure. The formation of the structure is possible, due to the presence of the overlapping phenomenon [13] in binary solutions of amylopectin.

## Conclusions

The results of the quality tests of the hysteresis loops revealed, that AM solutions in solvents formed of a mixture of H_2_O and DMSO with lower water concentration, are rheologically-stable fluids and exhibit a shear-thickening phenomenon. The AM solutions in a solvent composed of H_2_O and DMSO at the ratio of 500/500, display complex rheological behaviours manifested by oscillatory changes of viscosity in the function of shear time (at a stable shear rate). Due to a small radius of gyration and low expansion coefficient, a deformation of the AM coil during shear is possible, which enables the accumulation of energy. The AP solutions are rheologically unstable fluids. The results demonstrate, that instability of the starch solutions stems from the phenomena of energy accumulation. The capability of chains to accumulate energy results from the conformation they adopt in the analyzed solutions.

## Supporting information

S1 Fig Autocorrelation functions for solutions of amylose. (EPS)

S2 Fig Autocorrelation functions for solutions of amylopectin. (EPS)

## References

[1] Eliasson AC. Starch in Food: Structure, Function and Applications Cambrigde: Woodhead Publishing Limited; 2004.

[2] Aberle T, Burchard W, Vorwerg W, Radosta S. Conformational Contributions of Amylose and Amylopectin to the Structural Properties of Starches from Various Sources. Starke. 1994; 46: 329–335.

[3] Aberle T, Burchard W. Starches in semidilute aqueous solution. Starke. 1997; 49: 215–224.

[4] Radosta S, Haberer M, Vorwerg W. Molecular characteristics of amylose and starch in dimethyl sulfoxide, Biomacromolecules 2001; 2: 970–978. 11710058 10.1021/bm0100662

[5] Bello-Perez LA, Roger P, Baud B, Colonna P. Macromolecular Features of Starches Determined by Aqueous High-performance Size Exclusion Chromatography, J. Cereal. Sci. 1998; 27: 267–278.

[6] Roy S, Banerjee S, Biyani N, Jana B, Bagchi B. Theoretical and computational analysis of static and dynamic anomalies in water-DMSO binary mixture at low DMSO concentration. J. Phys. Chem. B. 2011; 115: 685–692 10.1021/jp109622h 21186810

[7] Ghosh R, Banerjee S, Chakrabarty S, Bagchi B. Anomalous behavior of linear hydrocarbon chains in water-DMSO binary mixture at low DMSO concentration. J. Phys. Chem. B 2011; 115: 7612–7620 10.1021/jp110549h 21591704

[8] Ghosh S, Chattoraj S, Chowdhury R, Bhattacharyya K. Structure and dynamics of lyzozyme in DMSO-water binary mixture: fluorescence correlation spectroscopy. RSC Adv. 2014; 4: 14378–14384

[9] Das DK, Patra A, Mitra RK. Preferential solvation of lyzozyme in dimethyl sulfoxide/water binary mixture probed by terahertz spectroscopy. Biophysical Chemistry 2016; 216: 31–36 10.1016/j.bpc.2016.06.002 27372901

[10] Jordan RC, Brant DA. Unperturbed dimensions of amylase in binary water / dimethyl sulfoxide mixtures. Macromolecules. 1980; 13: 491–499.

[11] Cheetham NWH, Tao L. Amylose conformational transitions in binary DMSO/water mixtures, Carbohydr. Polym. 1998; 35: 287–295.

[12] Divers T, Balnois E, Feller JF, Spevacek J, Grohens Y. The influence of O-formylation on the scale of starch macromolecules association in DMSO and water. Carbohydr. Polym. 2007; 68: 136–145.

[13] De Vasconcelos CL, de Azevedo FG, Pereira MR, Fonseca JLC. Viscosity-temperature-concentration relationship for starch-DMSO-water solutions. Carbohydr. Polym. 2000; 41: 181–184.

[14] Han JA, Lim ST. Structural changes of corn starches by heating and stirring in DMSO measured by SEC-MALLS-RI system. Carbohydr. Polym. 2004; 55: 265–272.

[15] Chakraborty S, Sahoo B, Teraoka I, Gross RA. Solution properties of starch nanoparticles in water and DMSO as studied by dynamic light scattering. Carbohydr. Polym. 2005; 60: 475–481.

[16] Ch Yang, Meng B, Chen M, Liu X, Hua Y, Ni Z. Laser-light-scattering study of structure and dynamics of waxy corn amylopectin in dilute aqueous solution. Carbohydr. Polym. 2006; 64: 190–196.

[17] Lee JH, You SG, Kweon DK, Chung HJ, Lim ST. Dissolution behaviours of waxy maize amylopectin in aqueous-DMSO solutions NaCl and CaCl_2_. Food Hydrocoll. 2014; 35: 115–121.

[18] Dintzis FR, Bagley EB, Felker FC. Shear-thickening and flow-induced structure in a system of DMSO containing waxy maize starch. J. Rheol. 1995; 39(6): 1399–1409.

[19] Dintzis FR, Berhow MA, Bagley EB, Wu YV. Felker FC. Shear-thickening behaviour and shear-induced structure in gently solubilised starches. Cereal Chem. 1996; 73(5): 638–643.

[20] Chamberlain EK, Rao MA. Rheological properties of acid converted waxy maize starches in water and 90% DMSO/10% water. Carbohydr. Polym.1999; 40: 251–260.

[21] Kapoor B, Bhattacharya M. Dynamic and extensional properties of starch in aqueous dimethylsulfoxide. Carbohydr. Polym. 2000; 42: 323–335.

[22] Kapoor B, Bhattacharya M. Steady shear and transient properties of starch in dimethylsufoxide Carbohydr. Polym. 2001; 44: 217–231.

[23] Ptaszek P, Łukasiewicz M, Ptaszek A, Grzesik M, Skrzypek, Kulawska M. Viscoelastic properties of highly concentrated maize starch solutions in DMSO. Starke. 2011; 63: 181–189.

[24] Barnes HA. Thixotropy–a review. J. Nonnewton. Fluid Mech. 1997; 70: 1–33.

[25] Mewis J, Wagner NJ. Thixotropy. Advances in Colloid and Interface Science. 2009; 147–148: 214–227. 10.1016/j.cis.2008.09.005 19012872

[26] Quemada D. Rheological modelling of complex fluids. II. Shear thickening behaviour due to shear induced flocculation. The European Physical Journal Applied Physics. 1998; 3: 309–320.

[27] Quemada D. Rheological modelling of complex fluids. III. Dilatant behaviour of stabilized suspensions. The European Physical Journal Applied Physics. 1998; 2: 175–181.

[28] Quemada D. Rheological modelling of complex fluids. IV. Thixotropic and “thixoelastic” behaviour. Start-up and stress relaxation, creep tests and hysteresis cycles. The European Physical Journal Applied Physics. 1998; 5: 191–207.

[29] Macosko CW. Viscous Liquid in Rheology Principles, measurements and applications. New York: VCH Publishers; 1994.

[30] Teraoka I. Polymer Solutions: An Introduction to Physical Properties. New York: Wiley-Interscience; 2002

[31] De Kee D, Turcotte G. Viscosity of biomaterials. Chem. Eng. Commun. 1980; 6: 273–282.

[32] Ptaszek A. The role of characteristic times in rheological description of structure forming food additives. J. Food Eng. 2012; 111: 272–278.

[33] Honerkamp J, Weese J. Tikhonov regularization method for ill-posed problems. Continuum Mechanics Thermodynamics. 1990; 2: 17–30.

[34] Ptaszek A. Time-dependent phenomena as evidence for structure-forming properties of starches, Starke. 2014; 66: 326–336.

[35] Schuster HG. Deterministic Chaos: An Introduction. Weinheim: WILEY-VCH Verlag GmbH & Co. KGaA; 2005.

[36] Heineck ME, Cardoso MB, Giacomelli FC, da Silveira NP. Evidences of amylose coil-to-helix transition in stored dilute solutions, Polymer. 2008; 49: 4386–4392.

[37] Millard MM, Dintzis FR, Willett JL, Lavons JA. Light-scattering weights and intrinsic viscosities of processed waxy maize starches in 90% dimethyl sulfoxide and H_2_O. Carbohydrates. 1997; 74: 687–691.

[38] Yu S, Xu J, Zhang Y, Kopparapu NK. Relationship between intrinsic viscosity, thermal and retrogradation properties of amylose and amylopectin. Czech Journal of Food Science. 2014; 32: 514–520.

[39] Ghosh A, Badiger MV, Tapadia PS, Ravi Kumar V, Kulkarni BD. Characterization of chaotic dynamics–I: dynamical invariants of sheared polymer solutions. Chem. Eng. Sci. 2001; 56: 5635–5642.

